# Hepatic and renal cellular cytotoxic effects of heparin-coated superparamagnetic Iron oxide nanoparticles

**DOI:** 10.1186/s40824-021-00241-7

**Published:** 2021-11-04

**Authors:** Yong Hwa Hwang, Youn-Jung Kim, Dong Yun Lee

**Affiliations:** 1 Department of Bioengineering, College of Engineering, and BK21 PLUS Future Biopharmaceutical Human Resources Training and Research Team, Institute of Nano Science & Technology (INST), Hanyang University, Seoul, 04763 Republic of Korea; 2 Department of Marine Science, College of Natural Sciences, Incheon National University, Incheon, 22012 Republic of Korea; 3 Research Institute of Basic Sciences, Incheon National University, Incheon, 22012 Republic of Korea; 4 Elixir Pharmatech Inc., Seoul, 04763 Republic of Korea

**Keywords:** Negative contrast agents, Superparamagnetic iron oxide (SPIO), Heparin, Dextran, Cytotoxicity

## Abstract

**Background:**

Superparamagnetic iron oxide (SPIO) nanoparticles have been widely used in several biomedical engineering in vivo. Although various surface modifications have been made to these non-biodegradable nanoparticles to make them more biocompatible, their toxic potential still remains a major concern.

**Method:**

In this study, we newly developed unfractionated heparin (UFH)-coated and low molecular weight heparin (LMWH)-coated SPIO nanoparticles through surface modification engineering, which was compared with commercially available dextran-coated SPIO nanoparticles. Their toxicity such as cytotoxicity, single cell gel electrophoresis (SCGE) comet assay, intracellular reactive oxygen species (ROS) content and cellular apoptosis was evaluated to hepatic HepG2 and renal HK-2 cells.

**Results:**

When UFH-, LMWH- or dextran-coated SPIO nanoparticles were applied, they did not affect the viability of HepG2 cell. However, HK-2 cells were more sensitive to dextran-coated SPIO nanoparticles than others. In genotoxicity assay using SCGE comet, DNA tail moment values in the groups treated with dextran- and LMWH-coated SPIO nanoparticles significantly increased. However, UFH-coated SPIO nanoparticles was only significantly lowing DNA tail moment value. In addition, UFH-coated SPIO nanoparticles had lower cytotoxicity in HepG2 and HK-2 cells compared to dextran-coated SPIO nanoparticles, especially in terms of apoptosis and intracellular ROS production.

**Conclusions:**

Collectively, it is possible that UFH- coated SPIO nanoparticles can be used as alternative negative contrast agents.

## Background

In vivo imaging of therapeutic cells with MRI after transplantation has improved our understanding of cell fate and has increased the focus on cell-based investigations. For cellular tracking with MRI, specific contrast agents have been incorporated into cells using various methods, including endocytosis and mechanical approaches [1]. Superparamagnetic iron oxide (SPIO) nanoparticles have been used for various applications, including targeted drug/gene delivery, tissue engineering, and magnetic transfection as a T_2_ contrast agent [2–4]. Current MRI protocols require direct labeling of cells with these contrast agents before transplantation. Once within cells, SPIO nanoparticles decrease signal intensity on T_2_-weighted and *T*_2_*-weighted images (called the ‘blooming effect’); they can generate notable signal loss with high sensitivity (single cell detection) during MRI at concentrations within the picogram range [1, 5]. SPIO nanoparticles vary in size, but they tend to aggregate into large clusters due to their surface-to-volume ratios and dipole-dipole interactions. When SPIO nanoparticles are injected into the bloodstream, it is ultimately eliminated from the blood circulation through opsonization with plasma proteins, which is followed by recognition and uptake by macrophages [6]. Therefore, the fate of SPIO nanoparticles is related to its physicochemical characteristics such as hydrodynamic size and surface charge [7, 8]. In addition, the sorts of surfaces coating materials and their functional group can affect in vivo pharmacokinetics and biodistribution of the nanoparticle [9–13].

During imaging of pancreatic islet cells after transplantation, Feridex or Resovist (dextran-coated SPIO nanoparticles) may be used [14, 15], but these show low cell labeling efficacy. Therefore, it is common to include a transfection agent such as poly-L-lysine (PLL), lipofectamine, or protamine sulfate to enhance endocytosis [16]. Unfortunately, these methods have shown cytotoxicity, and dextran-based SPIO contrast agents are not commercially available at present [17]. Resovist is stabilized by carboxydextran, while Feridex is coated with nonfunctional dextran. It is possible that Resovist can be uptake through the binding of cationic sites on the cellular membrane for cell labeling than Feridex because the carboxydextran is expected to be mainly in the deprotonated form under physiological conditions. For this reason, we previously developed heparin-based SPIO (HSPIO) nanoparticles and subsequently labeled cells, resulting in low toxicity [18–23]. Hydrophilic heparin, an anticoagulant agent, has highly negative charges because it is a heterogeneous group of anionic glycosaminoglycans. Like Resovist, therefore, the heparin-coated SPIO nanoparticles can be sued for cell labeling. According to heparin purification process, it was categorized by unfractionated heparin (UFH; 13,000-18,000 Da) and low-molecular-weight-heparin (LMWH; 3500-4500 Da). Therefore, it is possible that different molecular weight of heparin has different behavior. For example, LMWH binds less avidity to plasma proteins and increase bioavailability compared with UFH in vivo [24]. Even though we confirmed cell viability after HSPIO treatment in each study, toxicity issues in vicinity tissues after transplantation remain a major concern for cell-based therapy and for further clinical applications. Therefore, in this study, we evaluated in vitro hepatic and renal cytotoxic effects (including viability, inflammation, oxidative stress, and apoptosis) for heparin-based SPIO nanoparticles.

## Materials and methods

### SPIO preparation

Dextran-, UFH- and LMWH-coated SPIO nanoparticles were synthesized as described in previous reports [18, 19]. Briefly, SPIOs were synthesized via co-precipitation with FeCl_2_•4H_2_O (185 mg; Sigma, St. Louis, MO, USA) and FeCl_3_•6H_2_O (500 mg; Sigma). They were dissolved in deoxygenated distilled water (30 ml), and ammonium hydroxide solution (NH_4_OH, 7.5 ml) was added under nitrogen (N_2_) gas while stirring for 30 min. After removing unnecessary salt in the solution, 250 mg of either dextran (m.w. 40 kDa; Sigma), UFH, or LMWH (Nanjing King-Friend Biochemical Pharmaceutical Co., Ltd., Nanjing, China) was added to 20 ml of distilled water. Each solution was stirred for 2 h at room temperature and then sonicated (200 W; VCX-500 Ultrasonic Processor; Sonics & Materials, Inc., Newtown, CT, USA) for 60 min. The supernatant was heated at 80 °C for 1 h and applied to a magnetic field for 6 h. Finally, the solutions were centrifuged to remove aggregated particles at 4000 rpm for 10 min and stored at 4 °C until use.

### Cell culture and SPIO treatment

The human hepatocellular carcinoma cell line HepG2 and the human kidney proximal tubular cell line HK-2 were cultured in DMEM (Gibco, Grand Island, NY, USA) with 10% fetal bovine serum (FBS; Gibco) and 1% penicillin/streptomycin at 37 °C under a humidified 5% CO_2_ atmosphere. The medium was renewed every 2 days. The dextran-, UFH- and LMWH-coated SPIO nanoparticles were added at various concentrations (2.5 μg/ml, 5.0 μg/ml, and 10.0 μg/ml) to cell culture medium. The control cells were cultured with the same volume of medium without SPIO nanoparticles.

### Cytotoxicity assay

The cytotoxicity assay involved a 3-(4,5-dimethylthiazol-2-yl)-2,5-diphenyl tetrazolium bromide (MTT; Sigma) colorimetric assay. HepG2 and HK-2 cells were seeded in 96-well culture plates at a density of 2 × 10^4^ cells/well in 100 μL of culture medium. After 24 h of incubation, the cells were treated in triplicate with dextran-, UFH- or LMWH-coated SPIO nanoparticles at various concentrations (2.5, 5.0, and 10.0 μg/ml) for 6 h and 24 h, respectively. The SPIO-treated cells were washed by PBS at least 3 times to remove the residual SPIO nanoparticles. Following the treatment, the cells were incubated with MTT for 4 h. The solution was then removed, and 200 μl of DMSO was added to each well to dissolve formazan crystals. After thoroughly mixing, the plate was read at 570 nm, and the survival rate was calculated with a FLUOstar Optima instrument (BMG LABTECH GmbH, Germany).

### Single cell gel electrophoresis (SCGE) comet assay

The SCGE assay was performed according to the Singh method, with slight modifications [25]. The treatment concentrations of dextran-, UFH- and LMWH-coated SPIO nanoparticles were determined based on the results of the MTT assays. The HepG2 and HK-2 cells were seeded in 12-well plates with 3 × 10^5^ cells/well. Dextran-, UFH-, and LMWH-coated SPIO nanoparticles were added to cells at concentrations of 2.5, 5.0, and 10.0 μg/ml (respectively) for 24 h. DMEM medium and benzo [a] pyrene (BaP) were used as a negative and positive control, respectively. After treatment, all cells were washed with medium and resuspended in DMEM to achieve a concentration between 2 × 10^5^ and 2 × 10^6^ cells/ml. The 100 μl cell suspensions were then immersed in 100 μl of 1% low-melting-point agarose and layered onto microscope slides pre-coated with 200 μl of 1% normal-melting-point agarose. Samples were spread using a coverslip. After solidification, the slides were immersed in cold, fresh lysis solution (2.5 M NaCl, 100 mM Na_2_EDTA, 10 mM Tris, 10% DMSO, 1% Triton X-100, pH 10) for 1.5 h. The slides were then placed into a horizontal gel electrophoresis tank filled with cold electrophoresis buffer (1 mM Na_2_EDTA and 300 mM NaOH, pH 13) for 20 min to allow DNA unwinding. Electrophoresis was performed in the same buffer at 25 V and 300 mA for 20 min, and samples were neutralized using 0.4 M Tris (pH 7.5). The slides were then stained with 50 μl of ethidium bromide (EtBr, 20 μg/ml) before analysis. Finally, the images were taken by fluorescence microscopy (Leitz DIAPLAN, Germany) with a 549 nm excitation filter and a 590 nm barrier filter. One hundred randomly selected cells were analyzed with Komet 5 software (Kinetics Imaging). We retained the parameters of the comet Olive tail moment (length between center of head and center of tail multiplied by tail DNA).

### Measurement of intracellular reactive oxygen species (ROS)

The generation of ROS was detected using 2,7-dichlorofluorescein-diacetate (DCF-DA; Sigma). HepG2 and HK-2 cells were seeded in 96-well culture plates at a density of 1 × 10^4^ cells/well and treated with dextran-, UFH-, or LMWH-coated SPIO nanoparticles at concentrations of 2.5, 5.0, or 10.0 μg/ml (respectively) for 24 h. Hydroxyl peroxide was used as a positive control. After SPIO treatment, the cells were washed and incubated with 0.02 μg/ml of DCF-DA at 37 °C for 30 min. The relative fluorescence intensity of the cell suspensions was measured using a FLUOstar Optima device (BMG LABTECH GmbH, Germany).

### Cell apoptosis by FACS

Dextran-, UFH- and LMWH-coated SPIO-treated cells were prepared at a concentration of 1 × 10^6^ cells/ml. After treatment with ribonuclease (200 μg/ml) at 37 °C for 1 h, the samples were stained with propidium iodide (PI, 50 μg/ml in PBS) for 15 min at room temperature. DNA content was measured by exciting PI using a FACScan cytometer (Becton-Dickinson, Franklin Lakes, NJ, USA), and WinMDI (version 2.9) software was used for analyzing the data.

### Statistical analysis

The experiment was used as the experimental unit. All values are presented as the mean ± S.E.M. All error bars represent the S.E.M. A result was considered statistically significant at *p* < 0.05. The statistical analysis was performed with ANOVA or with the Kruskal-Wallis test.

## Results

### Cellular damage caused by various SPIO nanoparticles

**Fig. 1:**
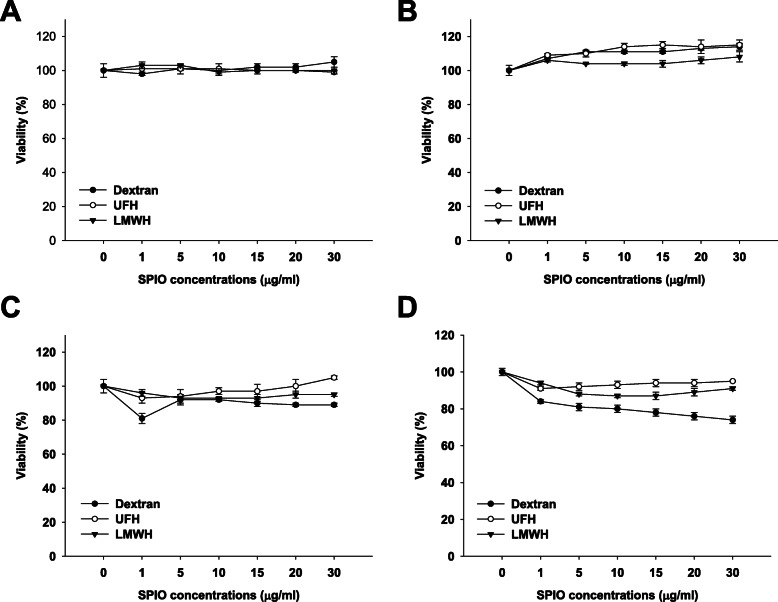
Cell viability test. MTT assay was performed after dextran-, UFH-, and LWMH-coated SPIO nanoparticles were applied to HepG2 cells for 6 h (**A**) and 24 h(**B**), and to HK-2 cells for 6 h (**C**) and 24 h (**D**)

In this study, we evaluated the cytotoxic effects of heparin-based SPIO nanoparticles on liver and kidney cells in vitro, using HepG2 and HK-2 cells. MTT assays showed different results according to SPIO type, concentration, treatment time, and cell type (Fig. 1). The cell viability in all groups treated with various concentrations of dextran-, UFH- and LMWH-coated SPIO nanoparticles was above 90% in HepG2 cells treated for 6 h (Fig. 1A) or 24 h (Fig. 1B). However, HK-2 cells were more sensitive to SPIO nanoparticles than HepG2 cells (Fig. 1C and D). All particles caused cytotoxicity in HK-2 cells after 24 h of treatment. Dextran-coated SPIO nanoparticles had the most cytotoxic result compared to other SPIO nanoparticles. Our heparin-coated (UFH and LMWH) SPIO nanoparticles reduced the cytotoxic effect compared to the dextran-coated SPIO nanoparticles.

### DNA damage caused by various SPIO nanoparticles: single cell gel electrophoresis (SCGE) comet assay

**Fig. 2:**
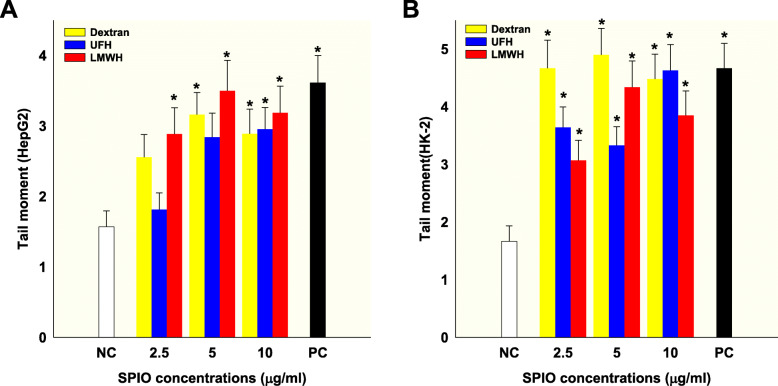
The SCGE comet assay. DNA damage caused by dextran-, UFH-, and LWMH-coated SPIO nanoparticles in HepG2 (**A**) and HK-2 (**B**) cells

Next, induction of DNA damage by dextran-, UFH- and LMWH-coated SPIO nanoparticles (concentration to 10 Fe μg/ml) in HepG2 and HK-2 cells was measured by the SCGE comet assay (Fig. 2). Compared with the control, tail moment values in the groups treated with dextran-coated SPIO nanoparticles (5.0 or 10.0 Fe μg/mL) significantly increased. LMWH-coated SPIO nanoparticles at any dose gave results significantly different to the control (*P* < 0.05) in HepG2 cells (Fig. 2A). However, treatment with UFH-coated SPIO nanoparticles was only significant at a dose of 10.0 Fe μg/mL. In the case of HK-2 cells, dextran-, UFH- and LMWH-coated SPIO nanoparticles caused significant DNA damage at every dose compared with the control (*P* < 0.05), but dextran-coated SPIO nanoparticles had the highest toxic effect (Fig. 2B).

### Relative ROS induction levels by various SPIO nanoparticles

**Fig. 3:**
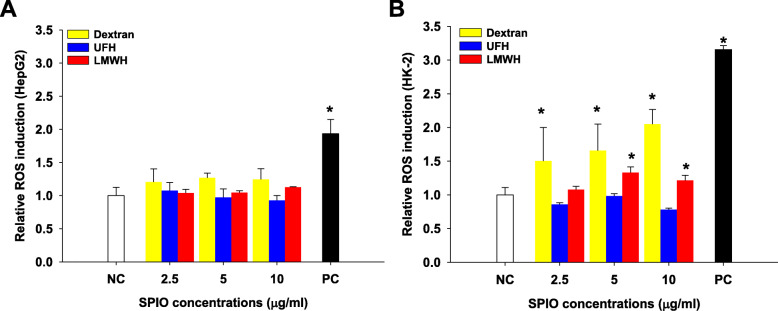
The intracellular reactive oxygen species assay. Relative ROS induction levels after dextran-, UFH-, or LMWH-coated SPIO nanoparticles were applied to HepG2 (**A**) and HK-2 (**B**) cells

We evaluated the generation of intracellular ROS by dextran-, UFH- and LMWH-coated SPIO nanoparticles in HepG2 and HK-2 cells, using the DCF-DA assay (Fig. 3). DCFH-DA itself has no fluorescence, and it can freely pass through cell membranes. After entering cells, it can be hydrolyzed by an esterase into DCFH, which can in turn be oxidized to DCF by ROS. DCF has fluorescence, and its intensity represents ROS levels. While ROS levels were not significantly increased in HepG2 cells (Fig. 3A), dextran and LMWH-coated SPIO nanoparticles induced dose-dependent ROS generation in HK-2 cells (Fig. 3B). However, UFH-coated SPIO nanoparticles did not affect ROS levels in either cell type.

### Apoptosis caused by various SPIO nanoparticles

Finally, we measured the cell apoptosis induced by dextran-, UFH- and LMWH-coated SPIO nanoparticles in HepG2 and HK-2 cells (Table 1). Apoptosis was not significantly different in HepG2 cells after treatment with dextran-, UFH-, and LMWH-coated SPIO nanoparticles. However, in HK-2 cells, dextran-coated SPIO nanoparticles affected apoptosis in a dose-dependent manner. UFH- and LMWH-coated SPIO nanoparticles did not significantly change apoptosis in HK-2 cells.

**Table 1:** Apoptosis caused by dextran-, UFH- and LMWH-coated SPIO nanoparticles in HepG2 and HK-2 cells

	Ratio of apoptosis (%)					
	HepG2			HK-2		
μg/mL	Dextran	UFH	LMWH	Dextran	UFH	LMWH
NC	1.33	1.33	1.33	0.83	0.83	0.83
2.5	1.77	1.71	1.66	1.6	0.85	0.69
5.0	2.03	1.81	1.54	2.38	0.97	1.58
10.0	2.25	2.24	2.13	5.68	0.96	1.58

## Discussion

Uncoated SPIO nanoparticles have very low solubility, causing precipitation that can impede blood vessels in a clinical setting. Therefore, to be used effectively in clinical trials, SPIO nanoparticles are coated with various materials such as dextran, citrate silicon, and PEGylated starch to improve biocompatibility and biodistribution [26]. However, the stability of these coatings with regard to the consequences of their breakdown in vitro or in vivo has not been thoroughly investigated. Commercially available contrast agents such as Ferridex, Resovist, Supravist, and Sinerem, which are coated with dextran or carboxyl dextran, indicate that dextran coatings are not strongly bound. Therefore, they are more prone to detachment, leading to aggregation and precipitation [2, 27]. In our previous studies, we fabricated heparin-based SPIO (HSPIO) nanoparticles by a thermal co-precipitation method and investigated their functions as MR contrast agents [18–23]. The hydrodynamic sizes of the dextran, LMWH, UFH-coated SPIO nanoparticles were 55.74 ± 6.22, 65.74 ± 8.48, and 62.5 ± 5.09 nm, respectively and their surface charge were − 6.26 ± 0.09, − 30.4 ± 0.14, and − 27.9 ± 0.57 mV, respectively. There were no significant differences among the three SPIO nanoparticle sizes. However, the Dextran-coated SPIO nanoparticles were significantly increased their size to 102.84 nm after 50 days compared to HSPIO nanoparticles. These HSPIO nanoparticles showed high stability and had higher saturated magnetization compared to dextran-based SPIO nanoparticles due to their high negative charges (over − 25 mV) [5]. Compared to dextran-coated SPIO nanoparticles, these HSPIO nanoparticles were more stably distributed in size and had branch-like structures (identified by long-term examination) [21]. Moreover, simple cell-labeling via endocytosis or a surface modification process was possible, allowing for successful cell tracking in vivo using MRI. Although we confirmed cell viability after HSPIO treatment in each study, toxicity issues remained a major concern, especially in the context of cell-based therapy. This is because the therapeutic effect can be significantly reduced when cells are exposed to toxic nanoparticles over a long period of time [28]. Toxic cellular effects include impaired mitochondrial activity, membrane leakage, and morphological changes that diminish therapeutic efficiency due to adverse effects on cell viability, proliferation, and metabolic activity [29]. When nanoparticle-treated cells are transplanted into the body, the risk of nanoparticles migrating through tissue or accumulating in surrounding tissue remains constant. This could trigger an immunological inflammatory response in the body while inducing other side effects [30, 31]. In this study, therefore, we evaluated the cytotoxic effects of heparin-based SPIO nanoparticles on liver and kidney cells in vitro, using HepG2 and HK-2 cells.

Most of the early studies on dextran-coated SPIO nanoparticles were conducted to understand the mechanism of cellular nanoparticle uptake [32, 33]. A variety of cells can be efficiently labeled with SPIO nanoparticles by simple incubation. It was discovered several years later that uncoated or dextran-coated SPIO nanoparticles could cause varying degrees of cell death, and were able to induce clear disruptions in the cytoskeleton of dermal fibroblasts [34, 35]. Further studies showed that the endocytosis-mediated cytotoxic effects were reduced by coating SPIO nanoparticles with different materials, such as lactoferrin and ceruloplasmin. Researchers demonstrated that the cytotoxic response could be modulated by specifically engineered particle surfaces [36]. Therefore, it became known that SPIO nanoparticle coating materials could influence cell-based therapy. Moreover, the surface coating of SPIO nanoparticles could significantly affect their fate and the extent of uptake because the surface coating drastically affects nanoparticle stability, aggregate size, and cellular interactions [4, 37].

In our previous studies, we chemically labeled the surface of pancreatic islet cells with HSPIO nanoparticles (for cell transplantation using a collagen membrane) at a concentration of 24 Fe μg/ml [19, 20]. Because the nanoparticles stably existed on the cell surface over 100 days [19] and their exposure to organ tissue (especially liver or kidney) was minimal, we lowered the SPIO nanoparticle concentration to 10 Fe μg/ml for intracellular uptake. For evaluation of cellular toxicity, in this study, induction of DNA damage by dextran-, UFH- and LMWH-coated SPIO nanoparticles in HepG2 and HK-2 cells was measured by using the SCGE comet assay. Detection of DNA damage (genotoxicity) is of high significance in toxicology and when assessing new pharmaceuticals. The SCGE comet assay is a versatile, sensitive, yet simple and economical technique used to measure DNA damage and repair in individual cells [25]. From the results, all the SPIO nanoparticles showed genotoxicity to HepG2 and HK-2 cell compared with the control group although all the SPIO nanoparticles showed different cytotoxicity to the cells. Especially, dextran-coated SPIO nanoparticles had the highest cytotoxic and genotoxic effect. The different cellular responsiveness might be related to different types of cellular membranes and basal mitochondrial oxidative capacity. Also, SPIO nanoparticles could induce intracellular ROS generation by activation of oxidative stress through nuclear condensation and chromosomal DNA fragmentation, then leading cells to apoptosis [38]. In addition, it might be related to the different expression of metabolic enzymes in different cells. It was reported that HepG2 cells secreted the liver specific plasma proteins, but were low the expression of metabolic enzymes, such as cytochrome P450 (CYP)-related enzymes [39]. Moreover, the expression of hepcidine, a hormone involved in the regulation of iron homeostasis, was not induced by exposure to dextran-coated SPIO nanoparticle [40]. Although ferritin (an iron storage protein complex) and ferroportin (an iron export molecule) were not altered following exposure to dextran-coated SPIO nanoparticles, transferrin-receptor 1 (TfR1) and hepcidin were significantly down-regulated in HepG2 cells [40]. Through these reports, we can suggest that the reason that the cellular effect by SPIO nanoparticles is not observed in HepG2 cells may be not due to the metabolism of SPIO nanoparticle, but rather due to the interaction between SPIO nanoparticle and the plasma protein secreted from HepG2 cells. Conversely, HK-2 cells have been reported to rapidly take up plasma protein in the medium [41]. Of course, because there are a few results on SPIO nanoparticle analysis and toxicity assay reported in HepG2 and HK-2 cells, it is necessary further studies to confirm this hypothesis.

On the other hand, the internalized SPIO nanoparticles are presumably degraded into free ions by hydrolyzing enzymes within the cell lysosomes. Free iron in the form of ferrous iron (Fe^2+^) can react with hydrogen peroxide and oxygen produced by the mitochondria to produce highly reactive hydroxyl radicals and ferric ions (Fe^3+^) via the Fenton reaction. Thus, iron is a source of ROS, and ROS could be dramatically reduced through administration of an iron chelator. Furthermore, it has been reported that compositional changes might occur over time in SPIO nanoparticles based on the oxidative state, affecting their shelf-life and degradation [42]. Based on these findings, Magnetite (Fe_3_O_4_) and maghemite (Fe_2_O_3_) can induce different cellular responses because of their ability to undergo oxidation/reduction reactions [43]. Its toxicity can be changed by coating magnetite particles in a way that results in fewer oxidative sites. In fact, we found that UFH-coated SPIO nanoparticles did not affect ROS levels in either cell type. In addition, dextran-coated SPIO nanoparticles caused significant cell death during macrophage exposure, and this was directly attributable to oxidative stress and the generation of free radicals [44, 45]. Based on these findings, we suggested that heparin coating can be much advantageous for cellular labeling with SPIO nanoparticle. The reason might be attributed to that heparin has a highly negative charge and a high tendency to bind to positively charged proteins and surfaces. More than 100 heparin-binding proteins have been identified, including numerous plasma proteins, cytokines, chemokines, and other small, biologically active molecules (not to mention endothelial cells themselves) [46–48]. Although most clinical usage of heparin is for its anticoagulant properties, its binding can interrupt numerous other biological pathways [49]. Heparin complexes with free hemoglobin itself and blocks the activity of free radicals (including ROS) [50]. Small amounts of heparin enhance the antioxidant activity of superoxide dismutase, and heparin sulfate proteoglycans tether superoxide to cell surfaces, contributing to the inhibition of free radicals in tissue injury. In terms of these characteristics of heparin, UFH-coated SPIO nanoparticles could have great potential for cell-based therapies and for MRI contrast agents lacking toxicity. In fact, we found that UFH-coated SPIO nanoparticles reduced cytotoxicity in HepG2 and HK-2 cells compared to LMWH-coated SPIO nanoparticles. Also, oxidative stress was lower in cells treated with UFH-coated SPIO nanoparticles. These results might be attributed to the larger molecular weight of UFH, which could affect more stable core coating to SPIO nanoparticle and the surface charge of UFH-coated SPIO nanoparticles.

## Conclusion

In this study, we evaluated the in vitro toxic effects of SPIO nanoparticles by measuring viability, genotoxicity, oxidative stress, and apoptosis. Conventional dextran-based SPIO nanoparticles initially appear stable, but they may eventually break down into an unfavorable product or exhibit exposed iron oxide cores. UFH-coated SPIO nanoparticles reduced cytotoxicity in HepG2 and HK-2 cells compared to LMWH-coated or dextran-coated SPIO nanoparticles. This suggests that UFH-coated SPIO nanoparticles could have great potential for cell-based therapies and for MRI contrast agents lacking toxicity.

## Data Availability

All data generated or analyzed during this study are included in this published article.

## Abbreviations

SPIO: Superparamagnetic iron oxide
UFH: Unfractionated heparin
LMWH: Low molecular weight heparin
MRI: Magnetic resonance imaging
PLL: Poly-L-lysine
HSPIO: Heparin-based SPIO
MTT: 3-(4,5-dimethylthiazol-2-yl)-2,5-diphenyl tetrazolium bromide
SCGE: Single cell gel electrophoresis
ROS: Reactive oxygen species
DCF-DA: 2,7-dichlorofluorescein-diacetate

## References

[1] Bulte JW, Kraitchman DL (2004). Iron oxide MR contrast agents for molecular and cellular imaging. NMR Biomed.

[2] McCarthy JR, Weissleder R (2008). Multifunctional magnetic nanoparticles for targeted imaging and therapy. Adv Drug Deliv Rev.

[3] Salgueirino-Maceira V, Correa-Duarte MA (2007). Increasing the complexity of magnetic core/shell structured nanocomposites for biological applications. Adv Mater.

[4] Gupta AK, Naregalkar RR, Vaidya VD, Gupta M (2007). Recent advances on surface engineering of magnetic iron oxide nanoparticles and their biomedical applications. Nanomedicine-Uk..

[5] Yang C-Y, Tai M-F, Chen S-T, Wang Y-T, Chen Y-F, Hsiao J-K (2009). Labeling of human mesenchymal stem cell: Comparison between paramagnetic and superparamagnetic agents. J Appl Phys.

[6] Gustafson HH, Holt-Casper D, Grainger DW, Ghandehari H (2015). Nanoparticle uptake: the phagocyte problem. Nano Today.

[7] Kumar A, Pandey AK, Singh SS, Shanker R, Dhawan A (2011). Cellular uptake and mutagenic potential of metal oxide nanoparticles in bacterial cells. Chemosphere..

[8] Singh R, Pantarotto D, Lacerda L, Pastorin G, Klumpp C, Prato M (2006). Tissue biodistribution and blood clearance rates of intravenously administered carbon nanotube radiotracers. Proc Natl Acad Sci U S A.

[9] Hwang YH, Lee DY (2012). Magnetic resonance imaging using heparin-coated superparamagnetic iron oxide nanoparticles for cell tracking in vivo. Quantitative Imaging Med Surg.

[10] Lechanteur A, Furst T, Evrard B, Delvenne P, Hubert P, Piel G (2016). PEGylation of lipoplexes: the right balance between cytotoxicity and siRNA effectiveness. Eur J Pharm Sci.

[11] Shen J, Kim HC, Su H, Wang F, Wolfram J, Kirui D (2014). Cyclodextrin and polyethylenimine functionalized mesoporous silica nanoparticles for delivery of siRNA cancer therapeutics. Theranostics..

[12] Nam K, Jung S, Nam JP, Kim SW (2015). Poly (ethylenimine) conjugated bioreducible dendrimer for efficient gene delivery. J Control Release.

[13] Xiao K, Li Y, Luo J, Lee JS, Xiao W, Gonik AM (2011). The effect of surface charge on in vivo biodistribution of PEG-oligocholic acid based micellar nanoparticles. Biomaterials..

[14] Tai JH, Foster P, Rosales A, Feng B, Hasilo C, Martinez V (2006). Imaging Islets Labeled With Magnetic Nanoparticles at 1.5 Tesla. Diabetes.

[15] Evgenov NV, Medarova Z, Pratt J, Pantazopoulos P, Leyting S, Bonner-Weir S (2006). In vivo imaging of immune rejection in transplanted pancreatic islets. Diabetes..

[16] Arbab AS, Yocum GT, Wilson LB, Parwana A, Jordan EK, Kalish H (2004). Comparison of transfection agents in forming complexes with ferumoxides, cell labeling efficiency, and cellular viability. Mol Imaging.

[17] Berman SMC, Walczak P, Bulte JWM (2011). Tracking stem cells using magnetic nanoparticles. Wires Nanomed Nanobi.

[18] Hwang YH, Jeong MJ, Kim MJ, Kim JK, Lee DY (2017). Enhancement of T2-weighted MR contrast using heparin for cell tracking in vivo. J Ind Eng Chem.

[19] Hwang YH, Kim MJ, Lee DY (2017). MRI-sensitive contrast agent with anticoagulant activity for surface camouflage of transplanted pancreatic islets. Biomaterials..

[20] Jung MJ, Lee SS, Hwang YH, Jung HS, Hwang JW, Kim MJ (2011). MRI of transplanted surface-labeled pancreatic islets with heparinized superparamagnetic iron oxide nanoparticles. Biomaterials..

[21] Lee JH, Jung MJ, Hwang YH, Lee YJ, Lee S, Lee DY (2012). Heparin-coated superparamagnetic iron oxide for in vivo MR imaging of human MSCs. Biomaterials..

[22] Jin SM, Oh SH, Oh BJ, Shim W, Choi JM, Yoo D (2015). Feasibility of islet magnetic resonance imaging using ferumoxytol in intraportal islet transplantation. Biomaterials..

[23] Mettler E, Trenkler A, Feilen PJ, Wiegand F, Fottner C, Ehrhart F (2013). Magnetic separation of encapsulated islet cells labeled with superparamagnetic iron oxide nano particles. Xenotransplantation..

[24] Cosmi B, Fredenburgh JC, Rischke J, Hirsh J, Young E, Weitz JI (1997). Effect of nonspecific binding to plasma proteins on the antithrombin activities of unfractionated heparin, low-molecular-weight heparin, and dermatan sulfate. Circulation..

[25] Nandhakumar S, Parasuraman S, Shanmugam M, Rao K, Chand P, Bhat B (2011). Evaluation of DNA damage using single-cell gel electrophoresis (comet assay). J Pharmacol Pharmacother.

[26] Sadeghiani N, Barbosa LS, Silva LP, Azevedo RB, Morais PC, Lacava ZGM (2005). Genotoxicity and inflammatory investigation in mice treated with magnetite nanoparticles surface coated with polyaspartic acid. J Magn Magn Mater.

[27] Jung CW (1995). Surface properties of superparamagnetic iron oxide MR contrast agents: ferumoxides, ferumoxtran, ferumoxsil. Magn Reson Imaging.

[28] Huang DM, Chung TH, Hung Y, Lu F, Wu SH, Mou CY (2008). Internalization of mesoporous silica nanoparticles induces transient but not sufficient osteogenic signals in human mesenchymal stem cells. Toxicol Appl Pharmacol.

[29] Yang CY, Hsiao JK, Tai MF, Chen ST, Cheng HY, Wang JL (2011). Direct labeling of hMSC with SPIO: the long-term influence on toxicity, chondrogenic differentiation capacity, and intracellular distribution. Mol Imaging Biol.

[30] Mahmoudi M, Hofmann H, Rothen-Rutishauser B, Petri-Fink A (2012). Assessing the in vitro and in vivo toxicity of superparamagnetic iron oxide nanoparticles. Chem Rev.

[31] Mahmoudi M, Simchi A, Imani M, Shokrgozar MA, Milani AS, Hafeli UO (2010). A new approach for the in vitro identification of the cytotoxicity of superparamagnetic iron oxide nanoparticles. Colloids Surf B Biointerfaces.

[32] Weissleder R, Cheng HC, Bogdanova A, Bogdanov A (1997). Magnetically labeled cells can be detected by MR imaging. J Magn Reson Imaging.

[33] Moore A, Weissleder R, Bogdanov A (1997). Uptake of dextran-coated monocrystalline iron oxides in tumor cells and macrophages. J Magn Reson Imaging.

[34] Berry CC, Wells S, Charles S, Aitchison G, Curtis ASG (2004). Cell response to dextran-derivatised iron oxide nanoparticles post internalisation. Biomaterials..

[35] Gupta AK, Berry C, Gupta M, Curtis A (2003). Receptor-mediated targeting of magnetic nanoparticles using insulin as a surface ligand to prevent endocytosis. IEEE Transactions Nanobiosci.

[36] Gupta AK, Curtis AS (2004). Lactoferrin and ceruloplasmin derivatized superparamagnetic iron oxide nanoparticles for targeting cell surface receptors. Biomaterials..

[37] Raynal I, Prigent P, Peyramaure S, Najid A, Rebuzzi C, Corot C (2004). Macrophage endocytosis of superparamagnetic iron oxide nanoparticles: mechanisms and comparison of ferumoxides and ferumoxtran-10. Investig Radiol.

[38] Lin XL, Zhao SH, Zhang L, Hu GQ, Sun ZW, Yang WS (2012). Dose-dependent cytotoxicity and oxidative stress induced by “naked” Fe3O4 nanoparticles in human hepatocyte. Chem Res Chin Univ.

[39] Donato MT, Tolosa L, Gomez-Lechon MJ (2015). Culture and functional characterization of human hepatoma HepG2 cells. Methods Mol Biol.

[40] Barisani D, Conte D (2002). Transferrin receptor 1 (TfR1) and putative stimulator of Fe transport (SFT) expression in iron deficiency and overload: an overview. Blood Cells Mol Dis.

[41] Zhao KW, Murray EJ, Murray SS (2017). HK2 proximal tubule epithelial cells synthesize and secrete plasma proteins predominantly through the apical surface. J Cell Biochem.

[42] Laurent S, Forge D, Port M, Roch A, Robic C, Vander Elst L (2008). Magnetic iron oxide nanoparticles: synthesis, stabilization, vectorization, physicochemical characterizations, and biological applications. Chem Rev.

[43] Karlsson HL, Gustafsson J, Cronholm P, Moller L (2009). Size-dependent toxicity of metal oxide particles--a comparison between nano- and micrometer size. Toxicol Lett.

[44] van den Bos EJ, Wagner A, Mahrholdt H, Thompson RB, Morimoto Y, Sutton BS (2003). Improved efficacy of stem cell labeling for magnetic resonance imaging studies by the use of cationic liposomes. Cell Transplant.

[45] Stroh A, Zimmer C, Gutzeit C, Jakstadt M, Marschinke F, Jung T (2004). Iron oxide particles for molecular magnetic resonance imaging cause transient oxidative stress in rat macrophages. Free Radic Biol Med.

[46] Hirsh J, Anand SS, Halperin JL, Fuster V (2001). Mechanism of action and pharmacology of unfractionated heparin. Arterioscler Thromb Vasc Biol.

[47] Gandhi NS, Mancera RL (2008). The structure of glycosaminoglycans and their interactions with proteins. Chem Biol Drug Des.

[48] Coombe DR (2008). Biological implications of glycosaminoglycan interactions with haemopoietic cytokines. Immunol Cell Biol.

[49] Lindahl U, Lidholt K, Spillmann D, Kjellen L (1994). More to "heparin" than anticoagulation. Thromb Res.

[50] Engelberg H (1999). Actions of heparin that may affect the malignant process. Cancer..

